# Composite Gel Polymer Electrolytes Based on Organo-Modified Nanoclays: Investigation on Lithium-Ion Transport and Mechanical Properties

**DOI:** 10.3390/membranes8030069

**Published:** 2018-08-24

**Authors:** Cataldo Simari, Ernestino Lufrano, Luigi Coppola, Isabella Nicotera

**Affiliations:** Department of Chemistry and Chemical Technologies, University of Calabria, 87036 Rende, CS, Italy; ernestino.lufrano@unical.it (E.L.); luigi.coppola@unical.it (L.C.)

**Keywords:** gel polymer electrolytes, composites, montmorillonite clays, lithium batteries, PFG-NMR, self-diffusion coefficient, blend polymers

## Abstract

Composite gel polymer electrolytes (GPEs) based on organo-modified montmorillonite clays have been prepared and investigated. The organo-clay was prepared by intercalation of CTAB molecules in the interlamellar space of sodium smectite clay (SWy) through a cation-exchange reaction. This was used as nanoadditive in polyacrylonitrile/polyethylene-oxide blend polymer, lithium trifluoromethanesulphonate (LiTr) as salt and a mixture of ethylene carbonate/propylene carbonate as plasticizer. GPEs were widely characterized by DSC, SEM, and DMA, while the ion transport properties were investigated by AC impedance spectroscopy and multinuclear NMR spectroscopy. In particular, ^7^Li and ^19^F self-diffusion coefficients were measured by the pulse field gradient (PFG) method, and the spin-lattice relaxation times (T_1_) by the inversion recovery sequence. A complete description of the ions dynamics in so complex systems was achieved, as well as the ion transport number and ionicity index were estimated, proving that the smectite clay surfaces are able to “solvatate” both lithium and triflate ions and to create a preferential pathway for ion conduction.

## 1. Introduction

Polymer electrolytes are regarded as one of the most promising candidates in advanced electrochemical applications, such as “smart” windows, displays, sensors, and more importantly, rechargeable lithium batteries [1,2,3,4]. For this last one, in particular, the research has focused for decades on gel-type membrane [5], generally achieved by immobilizing a liquid solution (for instance, a polar aprotic organic solvent or mixtures with a lithium salt) into a hosting polymeric matrix, such as poly(ethylene oxide) (PEO) and its derivatives (e.g., polyacrylonitrile (PAN), poly(vinylidene fluoride) (PVDF), poly(methyl methacrylate) (PMMA)) [6,7]. Respect to liquid electrolytes, in fact, gel polymer electrolytes (GPEs) are able to conjugate high ion conductivities with good mechanical strength, flexible geometry, reducing of liquid leaking and, thus, higher safety [8].

Owing to its ability to dissolve a large variety of salts, through interaction of its ether oxygen with cations, PEO has been one of the most extensively studied polymer used to prepare solid-state electrolytes, lighter, thinner, and safer for lithium-ion polymer batteries [9,10].

Thought, the low ionic conductivities at room temperature (10^−6^–10^−8^ S cm^−1^), the Li^+^ transference number lower than 0.5 and the poor mechanical strength, still hinder the large scale diffusion of PEO-based device. Conversely, PAN ensures an ionic conductivity of circa 10^−3^ S cm^−1^, satisfactory flame and mechanical resistances, but the dimensional stability of gels is poor [11,12]. After GPE preparation, in fact, a phase separation between the encapsulated electrolyte solution and the PAN matrix typically occurs, leading to a leakage problem and, thus, the passivation phenomena of the lithium electrode when in contact with the gel, as well as failure of the electrode/electrolyte contact both resulting in a dramatic reduction of the ionic conductivity.

One of the strategy undertaken to bypass the drawbacks is the blending method, according to which two or more polymers are mixed to obtain a blend electrolyte. As already probed [13,14,15,16] the method allows to easily control a large number of factors, directly affecting the thermal, mechanical and electrical properties of the final polymer electrolytes. By mixing PMMA and PVdF polymers, Nicotera and coworkers obtained a blend with remarkable improvement of mechanical stability respect to unblended polymers [17]. Helan et al. have been reported outstanding thermal stability up to 230 °C for PAN/PMMA blends, but with quite low ionic conductivity, of the order of 2 × 10^−7^ S cm^−1^ [18]. Very interesting electrical behavior and dimensional stability have been obtained by Choi et al. on PEO-PAN blend gel electrolytes, despite no evidence regarding mechanical resistance being provided [19].

An alternative approach for creating gel electrolyte system with improved mechanical properties and electrochemical performances foresees the incorporation of nanoscale organic/inorganic fillers within the polymer matrix [20]. The addition of SiO_2_ [21], Al_2_O_3_ [22], TiO_2_ [23], and other metal oxides [24,25] generally act as solid plasticizers, softening the polymer backbone and, thus, enhancing the segmental motion of the hosting polymer which, in turn, results in improved ion conductivity. 

Among inorganic fillers, layered nanoparticles based on clays have been actively investigated lately since they offer a large number of interesting properties such as high cation exchange capacity, large chemically active surface area, outstanding swelling ability, intercalation, catalytic activity, and high chemical and thermal stability. Finally, the properties of the smectite nanoclays can be tailored using simple chemical methods such as intercalation with organic or inorganic guest molecules. From the above, the dispersion of proper clay minerals within the polymer matrix could enhance the ionic conductivity improving at the same time the strength and heat resistance of the GPE.

Smectite clay with different particle sizes has been effectively tested as filler for the preparation of PEO nanocomposite electrolytes, demonstrating a discrete improvement of ionic conduction [26]. Kurian et al. [27] have shown that the surface modification of clay by ion exchange reactions with cationic organic surfactants such as alkyl amines, enhance the chemical affinity with the polymer matrix, leading to exfoliation of the clay particles and improving the gel’s strength. Organic montmorillonite (MMT) prepared by ion exchange with HTAB was dispersed in PAN polymer, obtaining a composite GPEs with improved thermal stability and ionic conductivity [28].

Despite the efforts, however, there is still the need to design a gel electrolyte able to guarantee adequate electrical performance without sacrificing mechanical strength and thermal resistance. In the present study, PAN/PEO blend (80:20 weight ratio) polymers were used in order to prepare nanocomposite GPEs with an organo-modified clay. Specifically, hydrated sodium calcium aluminum magnesium silicate hydroxide (SWy-2, Nanocor) was the natural montmorillonite/smectite clay selected since it is relatively inexpensive, widely available and has small particle size as well as it shows good intercalation capability. The organo-modification of the SWy-2 (org-SWy) was achieved by ion exchange reaction with hexadecyltrimethyl ammonium bromide (CTAB). The filler loading of org-SWy in the GPE was 10 wt % with respect to the polymers PAN/PEO. For the gel preparation, a mixture of ethylene carbonate (EC) and propylene carbonate (PC), with molar ratio EC:PC 1:0.4, was used as plasticizer, while lithium trifluoromethanesulfonate (LiTr) was the salt chosen.

In order to compare the effect of the clay on the gel properties, also not blended and filler-free GPE membranes were also prepared. 

All the GPEs were investigated by thermal (DSC), morphological (scanning electronic microscopy-SEM) and mechanical (DMA) analysis, while the ion transport studies were conducted by electrochemical impedance spectroscopy (EIS) and by multinuclear NMR spectroscopy. In particular, the ^1^H, ^7^Li, and ^19^F pulse-field-gradient (PFG) method was employed to obtain a direct measurement of the self-diffusion coefficients both of ions and solvents plasticizers (EC/PC), while the spin-lattice relaxation time (T_1_) was obtained by the inversion recovery sequence.

The combination of the electrochemical and NMR data has provided a wide description of the ions dynamics inside the so complex systems, as well as information on ion associations and interactions between polymers, filler and ions.

## 2. Materials and Methods 

### 2.1. Materials

Poly(ethylene oxide) (PEO, M.W. 5,000,000), polyacrylonitrile (PAN), lithium trifluoromethanesulfonate (LiCF_3_SO_3_ or LiTr, 99.95%), ethylene carbonate (EC, 98%), propylene carbonate anhydrous (PC, 99.7%), and hexadecyltrimethyl ammonium bromide (CTAB, 98%) were purchased from Sigma Aldrich, Milan, Italy and used as received.

Natural smectite Wyoming montmorillonite (SWy-2) has obtained from the Source Clay Minerals Repository, University of Missouri Columbia, MO, USA. The cation exchange capacity (CEC), measured by the Co(II) procedure, is equal to 80 mequiv. per 100 g of clay, charge density 0.6 e^−1^/unit cell (the unit cell is the Si_8_O_20_ unit) and particle size around 200 nm. The structural formula is Na_0.62_[Al_3.01_Fe(III)_0.41_Mg_0.54_Mn_0.01_Ti_0.02_](Si_7.98_Al_0.02_) O_20_(OH)_4_.

### 2.2. Synthesys of Organo-Modified Clay (Org-Swy)

SWy-2 were first fractioned to <2 μm by gravity sedimentation and purified by well-established procedures in clay science [29]. For the chemical modification, the cation exchange capacity of smectite clay has been exploited. CTAB (0.4 g) was dissolved in boiling deionized water until complete dissolution, then the resulting solution has been dropwise added, under vigorous stirring, to a dispersion of SWy-2 (1.0 g) in deionized water at 60 °C and left for 6 h to achieve the total cationic exchange. Finally, the mixture solution was separated by centrifugation, rinsed repeatedly with deionized water until Br− was completely removed, and dried for 24 h at 90 °C.

### 2.3. GPE Membrane Preparation

The solvent casting technique has been used to prepare both blended and not blended membranes, by immobilization of a lithium salt solution in a polymer matrix.

The required amounts of PAN and PAN-PEO (80/20 blend ratio) were dissolved in anhydrous dimetylformammide (DMF). The solution was stirred for several hours at 60 °C, until a homogeneous mixture was obtained and, after complete dissolution, the electrolyte solution was added. For the electrolyte solution, LiCF_3_SO_3_ was dissolved in a mixture of EC and PC with a fixed molar ratio (1:0.4). The lithium content, expressed as the ratio between the number of EC-PC moles and the LiTr moles (also O/Li ratio), was 10/1. Finally, the polymers/plasticizers [PAN:(EC-PC) and (PAN-PEO)/(EC-PC)] weight ratio was of 26:74.

For the nanocomposite GPEs, the appropriate amount of organo-modified clay has been added to DMF, mechanically stirred for 16 h and sonicated for 8 h to obtain a homogeneous dispersion. The dispersion was then added dropwise to the polymer solution, followed by further sonication and stirring. Here composite membranes with 10% of filler loading with respect to the polymer were prepared. The membranes were achieved by casting the solution on the aluminum plate at 50 °C overnight to favor the evaporation of DMF.

### 2.4. Characterization Techniques

NMR measurements were performed with a BRUKER AVANCE 300 Wide Bore spectrometer working at 116.6 MHz on ^7^Li, and 282.4 MHz on ^19^F, respectively. The employed probe was a Diff30 Z-diffusion 30 G/cm/A multinuclear with substitutable RF inserts. Spectra were obtained by transforming the resulting free-induction decay (FID) of single π/2 pulse sequences.

The pulsed field gradient stimulated-echo (PFG-STE) method [30] was used to measure the self-diffusion coefficients of lithium and triflate ions. The sequence consists of three 90° RF pulses (π/2 − τ_1_ − π/2 − τ_m_ − π/2) and two gradient pulses that are applied after the first and the third RF pulses, respectively. The echo is found at time τ = 2τ_1_ + τ_m_. Following the usual notation, the magnetic field pulses have magnitude g, duration δ, and time delay Δ. The FT echo decays were analyzed by means of the relevant Stejskal–Tanner expression:

$$I=I_{0}e^{-\beta D}$$

Here *I* and *I*_0_ represent the intensity/area of a selected resonance peak in the presence and in absence of gradients, respectively. β is the field gradient parameter, defined as β = [(γgδ)]^2^ (∆ − δ/3)]; *D* is the measured self-diffusion coefficient.

In these experiments, the used experimental parameters were: δ = 3 ms, time delay Δ = 30 ms, and the gradient amplitude varied from 350 to 1000 G cm^−1^. Based on the very low standard deviation of the fitting curve and repeatability of the measurements, the uncertainties in *D* values are estimated to about 3%.

Finally, longitudinal relaxation times (T_1_) of ^7^Li and ^19^F were measured by the inversion-recovery sequence (π – τ − π/2). All the NMR measurements were run by increasing temperature step by step from 20 to 80 °C, with steps of 10 °C, and leaving the sample to equilibrate for about 20 min at each temperature value.

From *D_Li_* and *D_F_* self-diffusion coefficients, σ*_NMR_* values were calculated according with the Nernst-Einstein equation:

$$\sigma_{NMR}=\frac{F^{2}c_{LiTr}}{RT}\left(D_{Li^{+}}+D_{F^{+}}\right)$$

Here, *F* is the Faraday constant, *R* is the molar gas constant, *T* is the temperature to which *D* has been measured and *c_LiTr_* is the salt concentration.

The ionic conductivity (σ, S cm^−1^) was measured by impedance spectroscopy recorded at OCV with an oscillating potential of 10 mV in the frequency range 0.1–1 × 10^6^ Hz using a PGSTAT 30 (MetrohmAutolab) potentiostat/galvanostat/FRA. GPEs were sandwiched between two disks of conductive carbon cloth, placed between two stainless steel electrodes and assembled in a homemade two-electrode cell. The impedance responses of the cell were analyzed using MetrohmAutolab NOVA software and the bulk resistance (*R_b_*) was extracted from the intercept of the low frequency signal in the Nyquist plot. The equation for calculating the conductivity is:

$$\sigma =\frac{l}{R_{b}*A}$$

where *l* is the thickness of the membrane and *A* is the area of the carbon cloth electrode.

Dynamic mechanical analysis (DMA) measurements were carried out on a Metravib DMA/25 analyzer equipped with a shear jaw for film clamping. Frequency sweep experiments were collected by subjecting a rectangular film to a dynamic strain of amplitude 10^-4^ in the range between 0.2 and 20 Hz. For temperature sweep (time cure) experiments a dynamic strain of amplitude 10^−4^ at 1 Hz was applied from 25 to 160 °C with a heating rate of 2 °C/min. A periodic sinusoidal displacement was applied to the sample, and the resultant force was measured. The damping factor, tan *d*, is defined as the ratio of loss (E′′) to storage (E′) modulus.

The thermal behaviors were investigated by Setaram 131 DSC. Samples were hermetically sealed and cooled from room temperature to −40 °C using liquid N_2_. Measurements were carried out from −30 °C up to 120 °C at the scan rate of 10 °C/min and purging nitrogen gas.

Finally, the membrane’s morphology was investigated by scanning electron microscopy (SEM, Cambridge Stereoscan 360, Santa Clara, CA, USA). To observe the membrane cross-sections, the samples were first frozen and fractured in liquid nitrogen, to guarantee a sharp fracture without modifications of the morphology, and then observed with SEM. The samples were sputter-coated with a thin gold film prior to SEM observation.

## 3. Results and Discussion

### 3.1. Morphological, Thermal, and Mechanical Characterizzation of the GPEs

**Figure 1:**
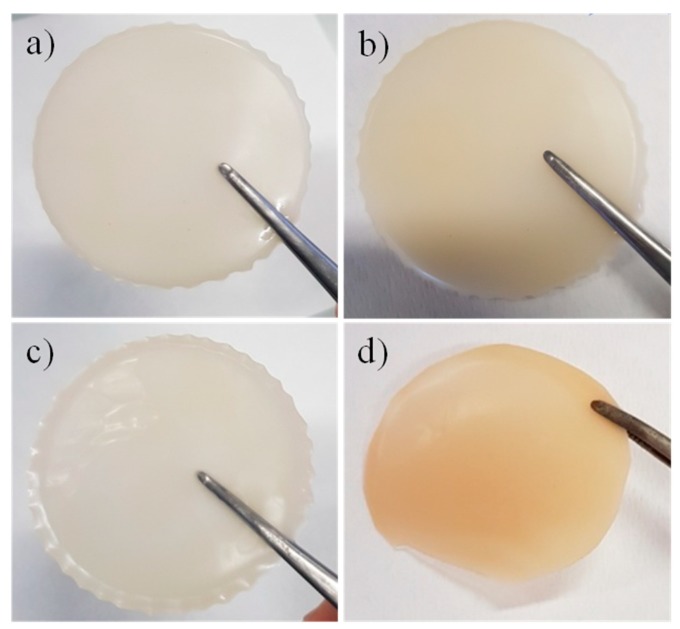
Photos of the prepared GPEs based on: (**a**) PAN, (**b**) PAN/org-SW, (**c**) PAN:PEO blend, and (**d**) PAN:PEO/org-SWy.

The organo-modification of the clay’s layers has as the main objective of favoring a good and homogeneous dispersion of the nanoparticles into the hosting matrix. For this purpose, hexadecyltrimethyl ammonium bromide was used as organophilic reagents: the quaternary ammonium group should allow an easy intercalation into the hydrophilic clay layers while the long alkyl chain should enhance the affinity between particles and polymer chains [31]. The photos of the four gel electrolytes prepared in this study are reported in Figure 1. They all appear opalescent, while the introduction of the org-SWy causes a slight yellowing of the resulting GPEs (Figure 1b,d). However, they are very dense and homogeneous, and there is no evidence of phase segregation between PAN and PEO polymers into the blended gels, indicating that the proposed method allows to obtain a homogenous and stable mixtures of polymers. Further, no clay particles crystals were observed, confirming that the chemical modification of the layers’ surface improves the clay/polymer interaction and, thus, highly homogeneous composite membranes, without formation of agglomerates or clusters, can be prepared.

**Figure 2:**
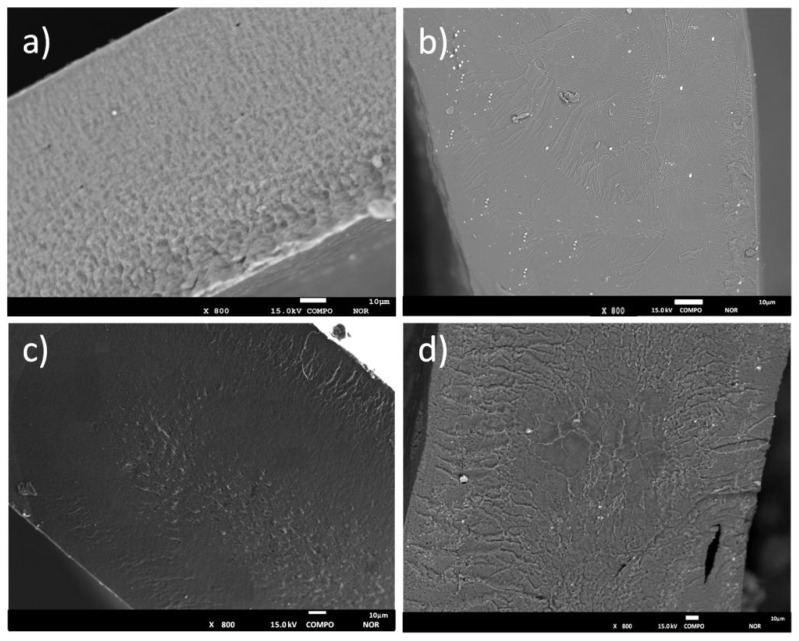
Cross-sectional SEM + BSE images of the GPEs based on: (**a**) PAN; (**b**) PAN/org-SWy; (**c**) PAN:PEO; and (**d**) PAN:PEO/org-SWy.

Scanning electron microscopy (SEM) coupled with BSE (backscattered electrons) was used to deeper investigate the morphology of the composite membranes. The BSE technique is generally used to detect contrast between areas with different chemical compositions (elements with high atomic number backscatter electrons more efficiently than light elements, appearing brighter in the image). By comparing the SEM-BSE images obtained on pristine PAN and PAN/org-SWy electrolytes, shown in Figure 2a,b, respectively, it clearly emerges that the presence of the filler particles severely affect the film morphology. The porous structure of the PAN based gel disappear in the composite gel, becoming a very dense membrane. Sporadic particle aggregations are also detectable, as expected if we take into account the large percentage of filler added into the polymer matrix (10 wt %). However, the average particles size of such aggregates is circa 500 nm, therefore, it can be stated that the nano-sized and homogeneous dispersion of clay layers was achieved in these composite GPEs. Concerning the blends (Figure 2c,d), SEM + BSE images give clear evidence that no phase separation occurs between the two polymers, as well as the presence of PEO allows the reduction of the number of nanosized aggregates in the composite blend electrolyte by virtue of a greater affinity between poly(ethylene oxide) chains and the org-SWy lamellae.

**Figure 3:**
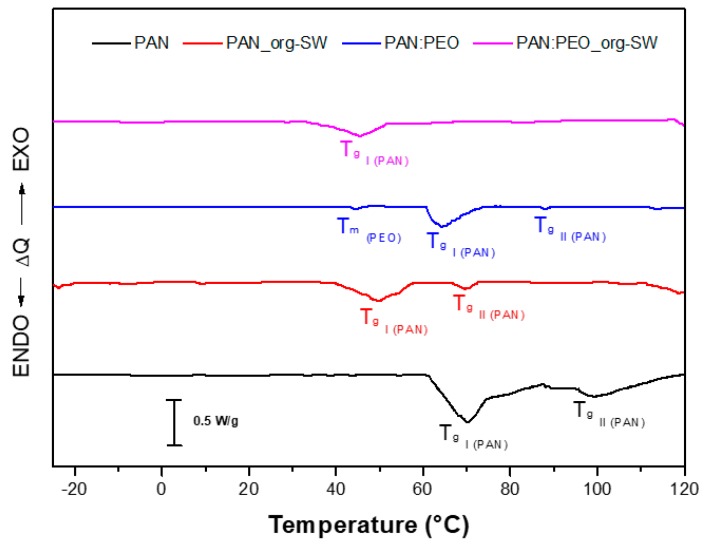
DCS thermograms of the GPEs membranes in the temperature range −30 °C up to 120 °C, with a scan rate of 10 °C min^−1^.

The analysis of the thermal properties of the prepared electrolytes has been carried out by DSC, and the thermograms collected in the temperature range between −30 and 120 °C are showed in Figure 3. For clarity it must be noticed that, in order to highlight the peaks, an enlarged scale was used.

The PAN-based gel shows two endothermic peak, the first one narrow, at about 71 °C (T_gI_), and the second broad peak at circa 100 °C (T_gII_). It was already demonstrated [32] that unoriented PAN has a “two-phase” morphology consisting of laterally-ordered and amorphous domains, both in a glassy state at room temperature, thus leading to two glass transition at 100 and 150 °C, respectively. In our films, the inclusion of EC/PC plasticizer lowers both T_g_ respect to pristine PAN, as a consequence of the reduction of the crystallites size [33]. The dispersion of org-SWy platelets leads to a large shift of the transitions of both laterally-ordered and amorphous domains (red line in the Figure 3), and also to a reduction of the peaks intensities, suggesting interactions between the organo-modified silicate layers and the polymer chains. It can be hypothesized that org-SWy particles increase the distance between polymer chains and, hence, diminish their capability to re-aggregate in glassy domains.

Focusing on blended electrolytes, in PAN:PEO films a small peak at 44 °C appears, corresponding to the typical temperature at which PEO crystalline domains becomes rubbery amorphous phase (T_m_ PEO). Finally, the nanocomposite blend electrolyte shows a single broad peak at 45 °C ascribed to the T_gI_ of PAN while disappear the T_m_ of PEO. The result can be explained in terms of larger chemical affinity between clay platelets and PEO chains, which reduces PEO re-crystallization and, at the same time, favors the dispersion of filler’s particles within the polymer matrix.

Concerning the mechanical properties of the GPEs systems, the measurements were performed by dynamic mechanical analysis, by using a shear jaw for films sample holder. It is worth pointing out that, generally, oscillatory rheological tests on typical GPEs are carried out by using a plate-plate geometry, while, in this case, due to the solid-like nature of our gels, a typical DMA configuration for thin films was used.

**Figure 4:**
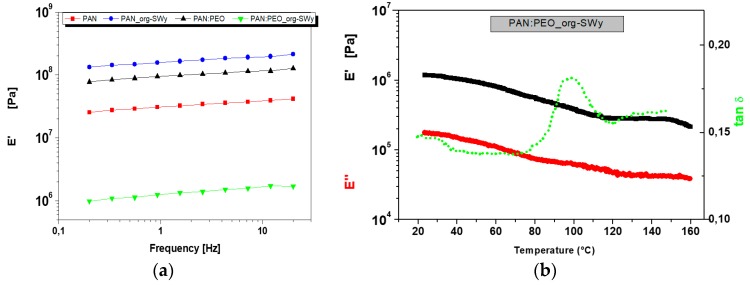
Frequency sweep at 25 °C of the different GPEs (**a**); and the temperature sweep test, from 20 °C to 160 °C for PAN:PEO/org-SWy electrolyte (**b**).

Figure 4a shows the storage modulus (E′) in the frequency range of 0.2–20 Hz measured at 25 °C: Except for one sample which will be discussed later, E′ shows values above 10^7^ Pa, significantly higher than other gels reported in the literature [34,35,36], and it reaches 10^8^ Pa upon inclusion of org-SWy lamellae in the PAN matrix, indicating an increase in the rigidity of the system. Blending PAN and PEO polymers also results in an enhanced storage modulus as a consequence of the increased overall crystallinity of the polymeric matrix. However, completely unexpected is the net reduction of the storage modulus of the composite blend PAN:PEO/org-SWy electrolyte to 10^6^ Pa. This evidence can be explained by taking into account the DSC data seen above. The inclusion of the clay into the polymer matrices prevents the reorganization of PAN and PEO chains into crystalline stacks, affecting the mechanical strength of the film but, at the same time, improves the flexibility of polymer chains, with important implications on the transport properties of this electrolyte gel. However, the temperature-sweep test shown in Figure 4b demonstrates that this composite still maintains the typical strong-gel behavior, likely due to the interactions between clay platelets and polymer chains. In fact, at least up to 160 °C, the storage modulus E’ exceeds significantly the loss modulus E′′, indicating that the gel responds elastically at small deformations and its microstructure is unchanged over this temperature range. The slight slope of the moduli is indicative of an evolution towards a “weak-gel” configuration, nonetheless, no crossover between the moduli occurs; therefore, the structure of the gel is preserved.

### 3.2. Transport Properties of Ions

**Figure 5:**
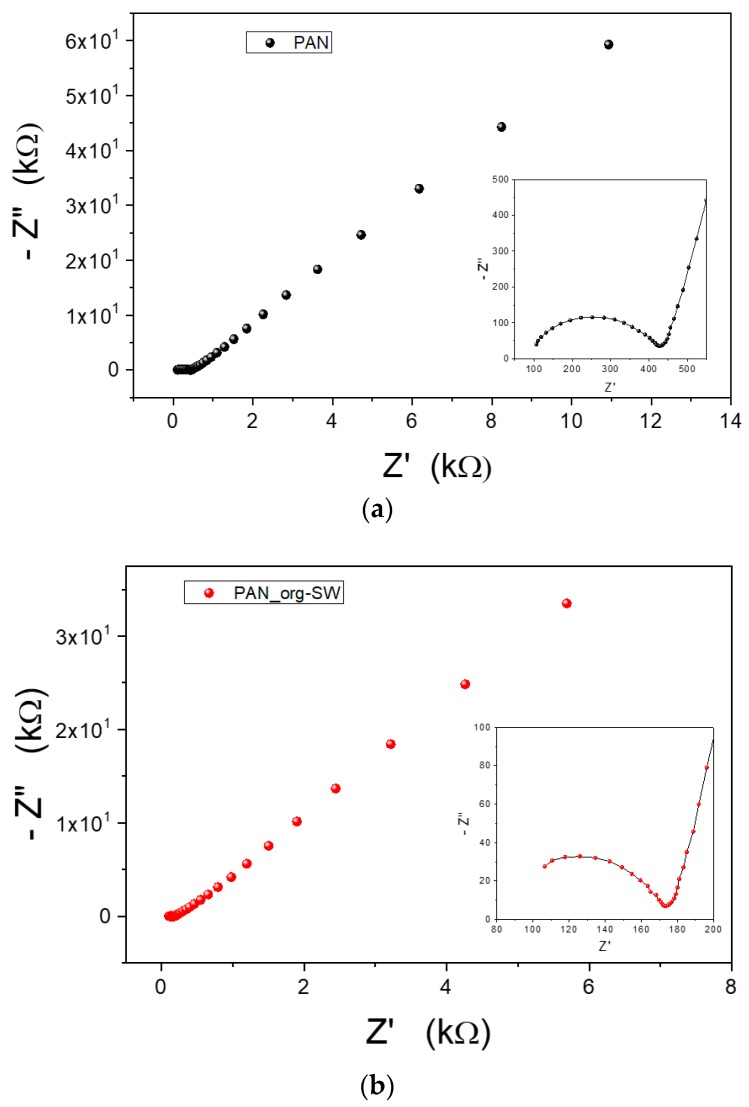
Nyquist plots of the impedance measured for PAN gel (**a**) and PAN/org-SWy nanocomposite gel (**b**).

The ionic conductivities of the prepared gel polymer electrolytes were investigated by EIS analysis. The impedance Nyquist plots of two representative GPEs are reported in Figure 5. The insets in each graph show an enlargement of the low resistance region, where the semicircle is achieved. In fact, the spectra show two well-defined regions: a semicircular region at high frequency range (attributed to ion conduction process in the bulk of the gel polymer electrolyte) followed by a straight line inclined at constant angle of circa 40° to the real axis at low frequency range related to the effect of blocking electrodes [37,38]. By comparing the spectra of PAN gels (Figure 5a) and of PAN/org-SWy nanocomposite (Figure 5b), we can notice that the semicircle of the nanocomposite appears as depressed, i.e., it is not completed in the frequency range used, although very high (1 MHz). This indicates that multiple processes and/or mechanisms of conduction simultaneously coexist [27]. A similar trend has been also observed in blended PAN:PEO/org-SWy electrolyte, even if less pronounced.

**Figure 6:**
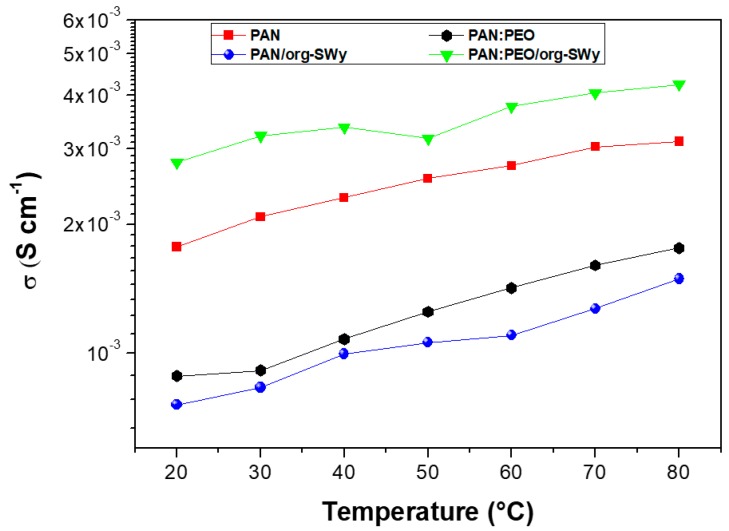
Temperature dependence of ionic conductivity for the gel polymer electrolytes investigated.

From the fitting of the semicircle in the high-frequency region, the electrolyte resistance was estimated and the ionic conductivity (σ) calculated according to the formula reported in the experimental and displayed in Figure 6. It clearly emerges that PAN-SWy nanocomposite gel is the less conductive electrolyte. Such an outcome can be explained by considering the changing of the gel morphology upon addition of the clay to the polymer matrix, as discussed above, which becomes dense, as well as more rigid (higher Young’s modulus). Therefore, the polymer chains experience lower flexibility, as well as a large reduction of liquid electrolyte mobility is expected by the decrease of the membrane porosity, both contributing to the reduction of the ion conduction.

Similar discussion can be made on the PAN:PEO blend gel, where the enhanced membrane rigidity caused by the increased number of crystalline domains of PEO significantly affects σ compared to the unblended PAN.

The best result was achieved by the addition of 10 wt % of organo-modified SWy in the PAN:PEO blend, which displays the highest ion conductivity over the whole temperature range, with a σ of almost 2.8 mS/cm at r.t. Comparing to similar GPEs reported in the literature, these conductivities are surely remarkable: e.g., they are two orders of magnitude higher than hybrid electrolytes composed of PEO and glass-ceramic particles (2.81 × 10^−2^ mS/cm) [26] and three orders higher than PEO containing conductive microsized particles (1 × 10^−3^ mS/cm) [39], while they are close to those reported by He et al. [31] for a PAN/organic montmorillonite system (2.23 mS/cm), even if, here, an electrolyte uptake of ca. 300% was needed, resulting in deterioration of the membrane stability. Accordingly, it can be stated that the PAN:PEO composite gels are able to guarantee good polymer chain flexibility together with outstanding mechanical and thermal resistance, making these systems particularly attractive as solid electrolytes for lithium batteries.

It is well known that the ionic conductivity obtained by EIS only refers to the mobility of charged species, with no possibility to distinguish between the cation and the anion. Conversely, NMR methods allow to discriminate and selectively investigate the mobility of Li^+^ and the corresponding counterion, confirming the effectiveness regarding the investigation of ions dynamics inside the complex systems, as well as information on ion associations and interactions between polymers, filler, and ions. Accordingly, NMR was used here to investigate the transport properties of both lithium cations and triflate anions, by detecting the ^7^Li and ^19^F spin-nuclei, respectively.

**Figure 7:**
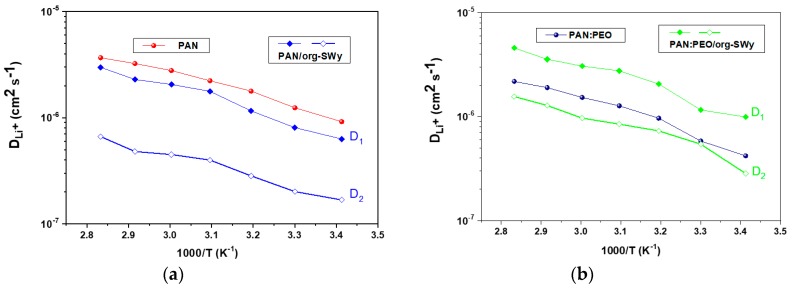
Arrhenius plots of ^7^Li self-diffusion coefficients from 20 to 80 °C measured on PAN-based electrolytes (**a**) and blended systems (**b**).

Figure 7 displays the lithium self-diffusion coefficients (*D_Li_*) measured on the GPEs’ membranes, both unblended (left) and blended (right), respectively. In agreement with the conductivity seen above, the addition of org-SWy to PAN reduces the lithium mobility while it has beneficial impact in the PAN:PEO blend. However, very interesting is the bi-exponential decay of the echo-signal obtained in both composite systems, observed also for the *D_F_* (diffusion values for ^19^F, not reported in the graph). This result indicates that two different mechanism for the diffusing species coexist as a consequence of the presence of the clay lamellae. The aluminosilicate platelets possess a fixed negative charge and the quaternary ammonium group of CTAB molecules was chosen as intercalating cation. Ions are solvated both from the clay layers (“lamellae-solvation”) and from the EC/PC solvents (“bulk-solvation”) and, of course, the polymers play their role in such coordination. 

Ions involved in the “bulk-solvation” show higher mobility (*D*_1_) respect to that one involved in the “lamellae-solvation” (*D*_2_).

**Figure 8:**
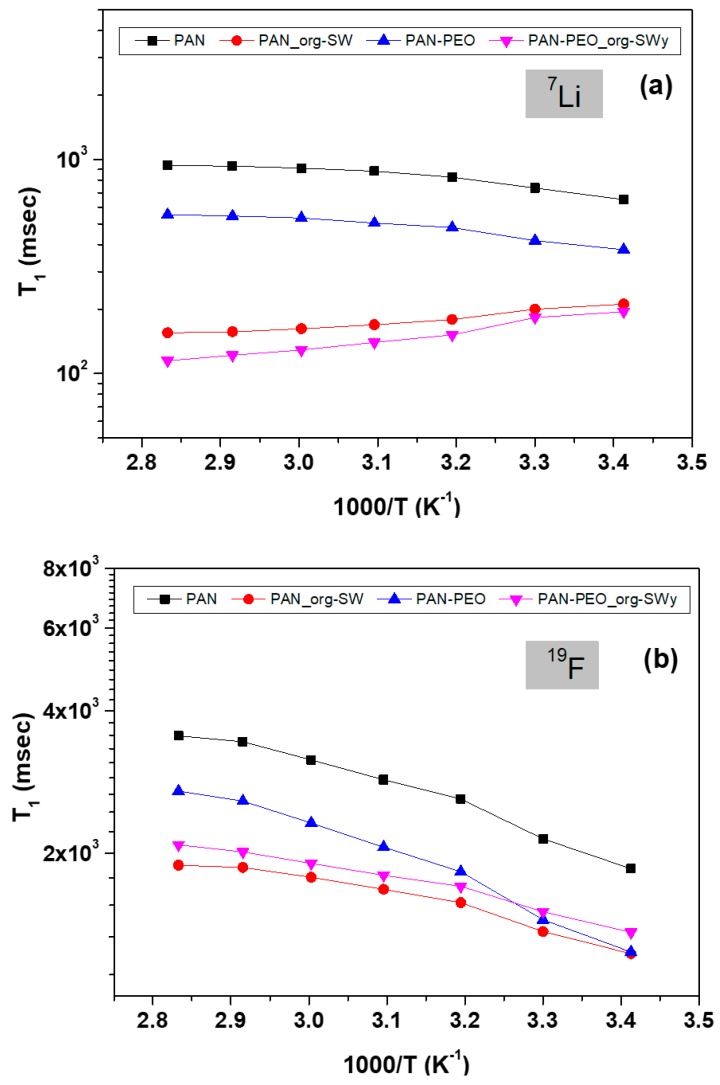
Arrhenius plot of ^7^Li (**a**) and ^19^F (**b**) spin-lattice relaxation time from 20 °C up to 80 °C.

Such a hypothesis was confirmed by the spin-lattice relaxation time (T_1_), which, compared to diffusion, reflects more localized motions, including both translation and rotation on a time scale comparable to the reciprocal of the NMR angular frequency (few nanoseconds). T_1_ quantifies the energy transfer rate from the nuclear spin system to the neighboring molecules (the lattice). The stronger the interaction, the quicker the relaxation (shorter T_1_). Figure 8 reports the Arrhenius plots of T_1_ measured on the different GPEs for ^7^Li and ^19^F, respectively. It is clear that the introduction of org-SWy particles produces a decrease of T_1_, both for ^7^Li and ^19^F. This outcome can be ascribed to the stronger overall interactions of the ions with the lattice, i.e., lithium ions interact with negative charged surface of the platelets, while counterions solvate the quaternary ammonium groups of the organo-surfactant. In other words, ions experience a lower degree of freedom resulting in shorter T_1_ values.

According to the Nernst-Einstein equation, conductivity values (σ*_NMR_*) were calculated from *D_Li_* and *D_F_* for the different GPEs and compared with the experimental ion conductivity (σ*_EIS_*) in Table 1 (for two representative gels). We need to consider that differently from σ*_EIS_*, σ*_NMR_* is affected not from the mobility of all species containing ^7^Li and ^19^F, including neutral ion pairs, and not only from the charged species. Therefore, it is not unusual for the NMR conductivity to be greater than the experimental σ, in particular when ion associations occurs. By considering the bi-exponentiality of both Li^+^ and F^−^ diffusion, and based on the hypothesis discussed above, we managed to calculate an average of *D*_1_ and *D*_2_ weighed with respect to the amount of filler added, i.e., 10 wt %. It is evident from the data reported that NMR conductivity values are always much higher than experimental ones suggesting the presence of a large number of ion pairing. This is also confirmed by the ionicity indices reported in Table 1 and computed as the ratio σ*_EIS_*/σ*_NMR_*.

PAN gel, our reference’s system, shows an ionicity close to 0.45. This suggests that 55% of Li^+^ and Tr^−^ exist as neutral ion pairs, which is typical for GPEs. The addition of filler particles into the blend increases the level of salt dissociation, likely due to the high dielectric constants of the charged organo-modified smectite clays that should also help to prevent the ionic association. Both phenomena leads to an ionicity index of 0.68 at r.t., which is a particularly high value for a double-ion solid-state electrolyte. Ionic association increases by increasing the temperature [11,39], therefore, the ionicity index decreases.

Finally, an important parameter for allowing a proper operation of the polymer electrolyte in real device is the lithium transport number ($t_{Li^{+}}$). It was calculated in this work according to the following equation and reported in Table 1:

$$t_{Li^{+}}=\frac{D_{Li^{+}}}{D_{Li^{+}}+D_{F^{-}}}$$

The PAN:PEO/org-SWy electrolyte shows a value of 0.68 at r.t., much higher than the PAN-gel and also the typical GPEs, for which values lower than 0.30 are generally reported [26,40,41,42,43]. GPEs with higher lithium transport number, i.e., ca. 0.55, has also been reported, but the ion conductivities are quite low [44]. The reasons of the improved $t_{Li^{+}}$ in our blend composite membrane can be multiple and synergistic: (i) the organo-clay particles have a plasticizing effect, lowering the cristallinity and, thus, improving the flexibility of polymer chains, favoring the Li^+^ transport through polymer segmental motions; and (ii) electrostatic interactions between the filler surface and lithium can create a preferential pathways for lithium conduction. 

## 4. Conclusions

Organo-modified smectite clay particles were prepared and dispersed into PAN and PAN:PEO blend polymers in order to prepare hybrid gel polymer electrolytes. Morphological studies proved that the procedure herein proposed allows to avoid phase separation between PAN and PEO as well as guarantee high nano-dispersion of the clay particles in the polymer matrix. The presence of the clay platelets strongly affected morphology, thermal and mechanical stability and electrochemical properties of the GPEs. In particular, outstanding behavior was displayed by the PAN:PEO/org-SWy membrane. ^7^Li and ^19^F NMR spectroscopy was successfully applied to get a complete description of the ions dynamics in so complex systems, probing as the smectite clay surfaces are able to “solvate” both lithium and triflate ions, preventing the ion pairing (as also confirmed by the high ionicity index) and creating preferential pathways for lithium conduction.

## Figures and Tables

**Table 1:** Comparison between σ*_EIS_* and σ*_NMR_*(in Ms cm^−1^), ionicity index and lithium transport number for PAN and PAN:PEO/org-SWy electrolytes.

T (°C)	PAN				PAN-PEO + 10% SW			
	σ*_EIS_*	σ*_NMR_*	Ionicity	*t_Li+_*	σ*_EIS_*	σ*_NMR_*	Ionicity	*t_Li+_*
20	1.77	3.92	0.45	0.40	2.79	4.31	0.68	0.68
30	2.08	5.02	0.41	0.41	3.22	4.74	0.65	0.67
40	2.31	6.96	0.33	0.41	3.37	7.84	0.53	0.59
50	2.56	8.19	0.31	0.43	3.17	9.07	0.45	0.56
60	2.74	9.68	0.28	0.44	3.77	9.82	0.48	0.56
70	3.03	11.1	0.27	0.43	4.05	11.20	0.42	0.57
80	3.12	11.8	0.27	0.45	4.24	12.33	0.38	0.58
